# The Effect of Changes in Magnetic Field and Frequency on the Vibration of a Thin Magnetostrictive Patch as a Tool for Generating Guided Ultrasonic Waves

**DOI:** 10.3390/s22030766

**Published:** 2022-01-20

**Authors:** Akram Zitoun, Steven Dixon, Mihalis Kazilas, David Hutchins

**Affiliations:** 1 Brunel Composites Centre, College of Engineering, Design and Physical Sciences, Brunel University London, London UB8 3PH, UK; akram.zitoun@brunel.ac.uk (A.Z.); mihalis.kazilas@brunel.ac.uk (M.K.); 2 School of Engineering, The University of Warwick, Coventry CV4 7AL, UK; D.A.Hutchins@warwick.ac.uk; 3 Department of Physics, The University of Warwick, Coventry CV4 7AL, UK

**Keywords:** magnetostriction, magnetic field, magnetic domains, vibration

## Abstract

A set of experiments was designed and conducted to investigate the vibrational ultrasonic response of a thin magnetostrictive patch bonded to a glass plate, with changes in static and dynamic magnetic fields applied to the patch. Such arrangements are often used as a means of generating guided waves in pipes or plates, by attaching a patch to a sample’s surface. The effect of varying the applied static and dynamic magnetic field’s amplitudes and directions and the frequency of the dynamic magnetic field was studied. It was demonstrated that the vibration of the magnetostrictive patch could be controlled and enhanced by optimizing the magnetic fields. It was also shown that for low-amplitude dynamic magnetic fields, Lorentz forces generated within the patch and the resonant frequency of the patch could also contribute to the enhancement of the vibration of the patch for low-amplitude fields. For high-amplitude dynamic magnetic fields, the magnetostriction effect can be the main transduction mechanism, which can be optimized for non-destructive testing and inspection purposes.

## 1. Introduction

Magnetostriction is a physical phenomenon that occurs in almost all ferromagnetic materials. It was first reported by James Joule in 1842 [1], and thereafter was investigated by other authors [2,3]. The term magnetostriction refers essentially to the coupling between the applied magnetic field and the strain or stress generated due to such a field. The direct effect (also called the Joule effect) occurs when the dimension of the sample changes due to the application of an external magnetic field. The inverse effect (the Villari effect) occurs when the magnetization of the ferromagnetic sample changes due to strain [4,5,6]. Other coupling concepts have also been investigated, including the Wiedemann effect [7], the Matteucci effect [8] and the magnetoimpedance stress dependent effect [9]. Magnetostriction is commonly observed when an external magnetic field is applied to a ferromagnetic material [10].

The magnetostrictive coefficient is usually written as *λ* and is defined as the ratio of the fractional change in length *δl* over the initial length *l*_0_, as shown in Equation (1):

(1) $$\lambda =\frac{\delta l}{l_{0}}$$

Atomic magnetic dipoles are associated with atoms located at specific locations within the crystal lattice of the ferromagnetic sample. These magnetic dipole moments tend to align in the same direction within a magnetic domain spontaneously, as this situation is energetically favorable [11]. In the absence of an external magnetic field, the magnetic domains within a ferromagnetic sample are oriented in a distribution that minimizes the external magnetic field [12,13,14]. When an external magnetic field is applied to the ferromagnetic material, the magnetic domains and the magnetic dipole moments start to align with the direction of the external applied magnetic field. This generates strain within the material due to the magnetic anisotropy in the crystal structure. The degree of strain induced depends on the specific sample properties, including material composition and microstructure and the shape of the sample [15].

**Figure 1:**
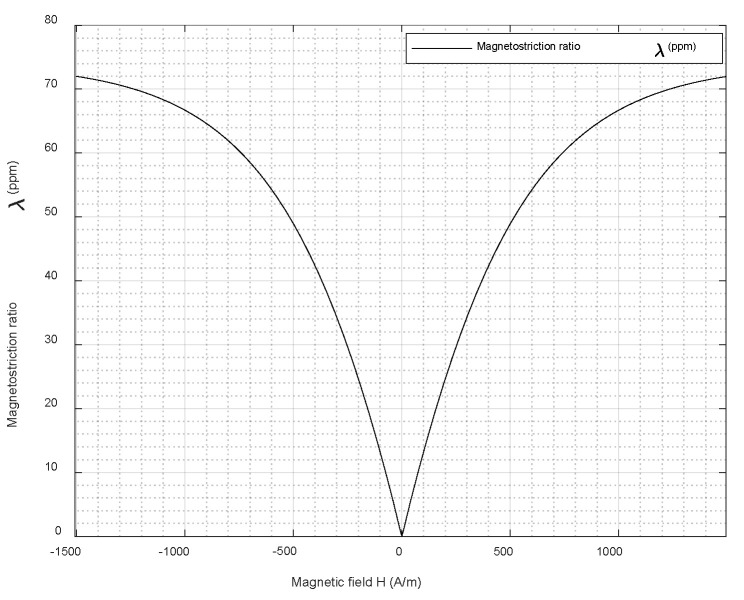
Magnetostriction curve for an iron–cobalt alloy obtained through modeling in Comsol.

For materials with polycrystalline structures, the magnetostriction ratio can be obtained using the following equation:

(2) $$\lambda =\frac{3}{2}\lambda_{100}(\alpha_{1}^{2}\beta_{1}^{2}+\alpha_{2}^{2}\beta_{2}^{2}+\alpha_{3}^{2}\beta_{3}^{2})+3\lambda_{111}(\alpha_{1}\alpha_{2}\beta_{1}\beta_{2}+\alpha_{2}\alpha_{3}\beta_{2}\beta_{3}+\alpha_{3}\alpha_{1}\beta_{3}\beta_{1})$$

where *λ*_100_ and *λ*_111_ are the magnetostriction coefficients along the <100> and <111> directions, respectively, and *α_i_* (*i* = 1, 2, 3) and *β_i_* (*i* = 1, 2, 3) are, respectively, the direction cosines relative to the field direction overlapping with the axis where the magnetization is saturated and the direction overlapping the axis following which the magnetostriction is measured [16,17]. As can be seen from Equation (2), there is a nonlinear relationship between the external applied magnetic field and the resultant strain, and this needs to be taken into consideration when designing a magnetostriction-based system. The nonlinear relationship between the applied field (*H*) and the magnetostriction ratio (*λ*) for an iron–cobalt alloy is illustrated in Figure 1.

The magnetostriction effect observed and the behavior of the material when a field is applied differs from one material to another [18,19,20,21]. As shown in Figure 1, the magnetostriction curve is symmetric in shape [22,23] and is obtained by slowly varying an externally applied magnetic field to a ferromagnetic specimen and capturing the change in dimensions. The *X*-axis can be represented by the magnetic flux density *B*, magnetic field strength *H* or magnetization *M*.

The relationship linking the magnetic properties and the stress-related response of the magnetostrictive material, as reported in [23,24,25,26,27], can be written as follows:

(3) $$\varepsilon =s^{\left(H\right)}\sigma +dH$$

(4) $$\sigma =c\varepsilon -d^{-1}H$$

(5) $$B=d^{T}\sigma +\mu^{\left(\sigma \right)}H$$

where *ε*, *σ* and *B* are strain, stress and magnetic field density matrices, respectively, and *s*^(*H*)^, *c* and *d* are the elastic compliance at a specific value of *H*, the stiffness and the piezomagnetic matrices, respectively. *d^T^* is the transpose matrix of *d*, *d*^−1^ is the inverse matrix of *d* and *µ*^(*σ*)^ is the permeability of the material measured at a specific value of the stress *σ*. The variables *s*^(*H*)^, *d* and *µ^σ^* are nonlinear, as they depend on the stress and the magnetic field. These equations can be used to design magnetostrictive thin films as either sensing or actuating elements. Equation (3) establishes the magnetostrictive element as an actuation system via the Joule effect, whereas Equation (5) establishes the magnetostrictive material as a sensing element via the Villari effect.

The magnetostriction phenomenon is widely used for different applications, such as the active element in ultrasonic transducers used to generate elastic waves for nondestructive testing and inspection [28,29,30,31], microelectromechanical systems or MEMS [29], positioning sensors [32], level-gauging sensors [33] and accurate actuation mechanisms [34]. 

Despite the fact that magnetostriction is a well-known physical property of ferromagnetic materials and that it has numerous applications within industry, some aspects of magnetostrictive behavior do not seem to have been reported fully, such as the effect of the variation of the excitation frequency and the direction and amplitude of the externally applied magnetic fields. The dynamic magnetic field driving frequency has been considered critical in designing the magnetostrictive patch [35], as ultrasonic guided wave application often requires the generation of a specific frequency of wave with a specific displacement of wave at the sample surface. Numerical modeling has established that different guided wave modes can be optimally selected and generated by modifying the coil geometry that generates the dynamic magnetic field and by optimizing the permanent or static magnetic field that magnetically biases the patch [36]. 

This experimental paper helps to explain and demonstrate the response of a thin patch due to magnetostriction from different orientations and relative amplitudes and excitation frequencies of dynamic and static magnetic fields. The experimental sample used here is a patch bonded to a large glass plate to generate ultrasonic guided waves in the plate [29], but the general findings of the forces generated at the patch can be applied to other types of guided waves where a patch may be applied to a pipe or a bar, for example.

## 2. Experimental Work 

### 2.1. Electromagnet and RF Coil Design Generating the Different Magnetic Fields 

**Figure 2:**
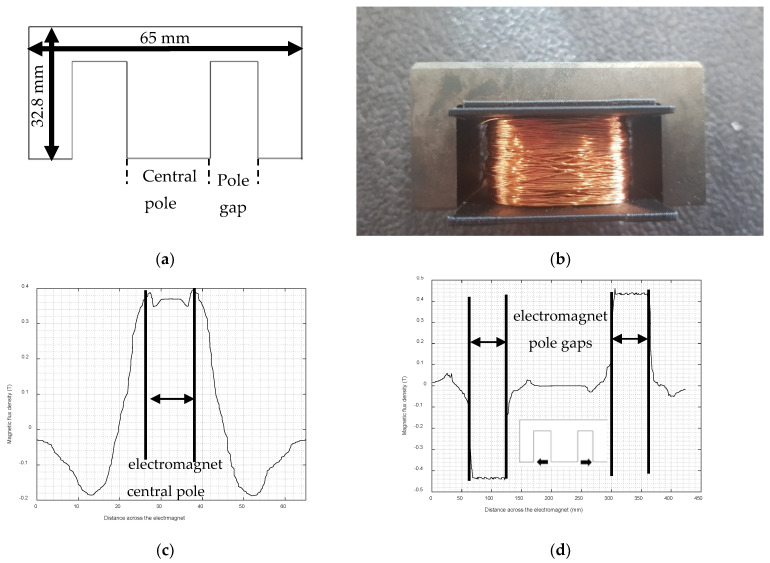
(**a**) The ferrite core used for the electromagnet; (**b**) photograph of the assembled electromagnet. Results of Comsol modeling for the magnetic flux density generated by the electromagnet are shown across (**c**) the central pole region and (**d**) in the two pole gaps.

An electromagnet was used to provide a bias magnetic field for the magnetostrictive patch, that was effectively a pseudostatic field compared to the dynamic magnetic field obtained from a different coil. The electromagnet used in these experiments contained an E-shaped ferrite core with dimensions 65 mm (length) × 32.8 mm (height) × 27.4 mm (width), as shown in Figure 2a,b. This shape enabled two directions of static magnetic field (B_s_) to be applied to the thin magnetostrictive patch that was bonded to the sample being tested. Placing the patch in the region under the central pole of the electromagnet allowed the static magnetic field to be applied perpendicularly to the surface, whilst an in-plane field would be generated within the pole gaps.

A COMSOL finite element model was developed to investigate the magnitude of the magnetic flux density generated by the electromagnet. The results are presented in Figure 2c for the area within the central pole region (where the field was predominantly perpendicular to the face of the electromagnet) and in Figure 2d for the pole gap region (where it was mainly parallel).

**Figure 3:**
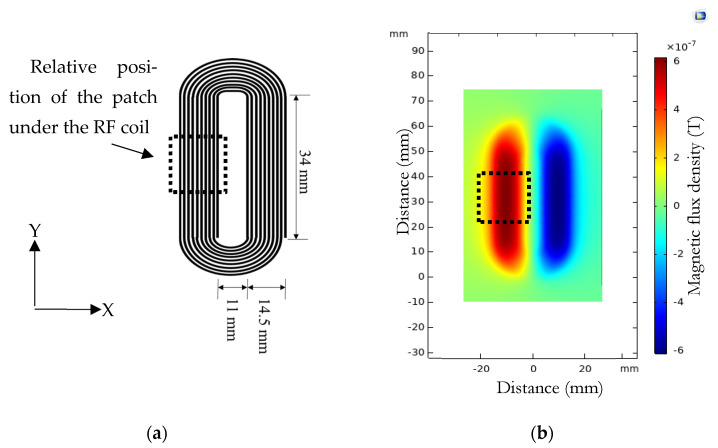
(**a**) The dimensions of the coil used to generate the dynamic magnetic field. (**b**) COMSOL model results for the dynamic magnetic field (B_d_) and the relative position of the magnetostrictive patch.

Two hundred turns of wire were used for the electromagnet coil so as to generate a magnetic flux density between the poles of up to 1 T. Modeling indicated that a current of 25 amps would be needed in the presence of a magnetostrictive patch, which altered the field from the free space value. The same current would produce a magnetic flux density of approximately 0.5 T if the patch was not present. This was because the patch had a high value of magnetic permeability, which would concentrate magnetic field lines and thus increase the flux density. The source of the dynamic magnetic field (B_d_) was a racetrack coil of 10 turns and a 0.5 mm spacing between turns, on a printed circuit board (PCB), and is shown in Figure 3a. The COMSOL model results shown in Figure 3b provide an understanding of the characteristics of the magnetic field generated by the rf coil and helped in the transducer design to optimize field generation in the required direction. The magnetostrictive patch had to be accommodated under this coil so that B_d_ was always applied in the direction parallel to the surface of the patch. The magnetostrictive patch used in the experiments was fabricated from a VACOFLUX 48 iron–cobalt alloy supplied by ^®^VACUUMSCHMELZE GmbH & Co., Hanau, Germany, with the dimensions and properties given in Table 1. These patches exhibit isotropic behavior when different static and dynamic fields are applied [29], as demonstrated by placing the patch under a coil and permanent and then rotate the system by 90° while monitoring the vibration behavior. It was shown that rotation had minimal effect on the vibration of the specimen.

### 2.2. Experimental Apparatus

The experiments were conducted to investigate the change in vibrational properties of the magnetostrictive patch in the three orthogonal (*X*, *Y*, *Z*) directions for variations in both the static magnetic field from the electromagnet (B_s_) and the RF coil (resulting in changes in the dynamic field B_d_).

**Figure 4:**
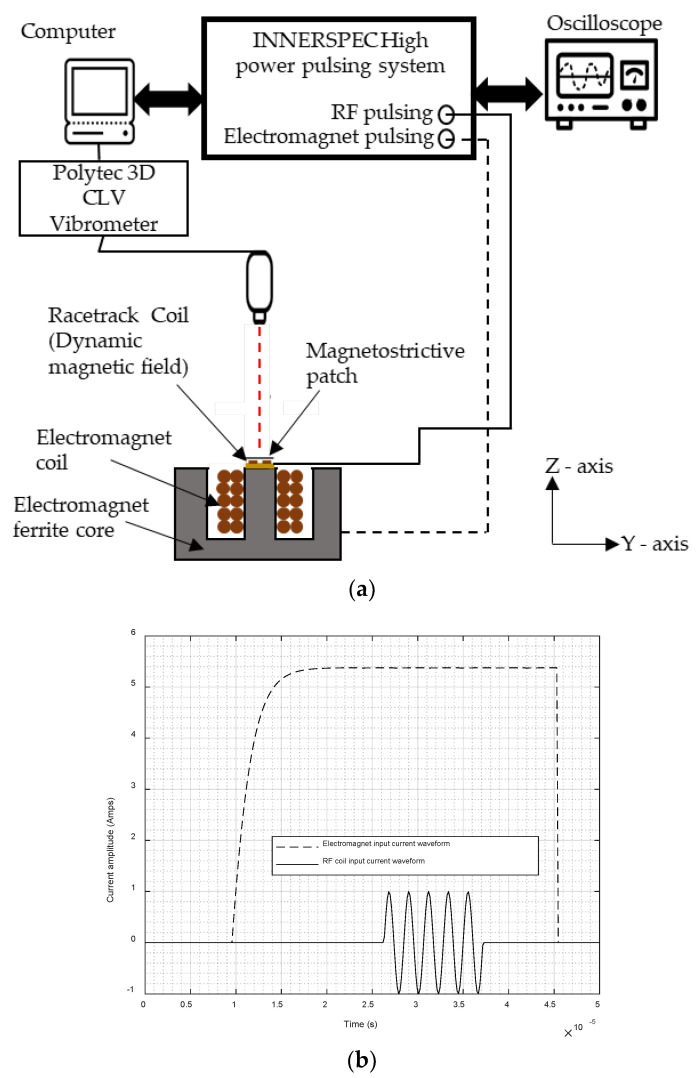
(**a**) Schematic diagram of the apparatus. (**b**) The nominal output from the dual electromagnet/rf coil driving system, showing both the electromagnet drive current and the 5-cycle tone-burst used to excite the rf pancake coil. Note that the rf coil was excited once the current to the electromagnet had stabilized.

A schematic diagram of the apparatus is shown in Figure 4a. The custom-designed Innerspec pulsing unit contained two subsystems, providing different outputs: an RF current pulsing unit connected to the RF PCB coil, which provided a nominal 5-cycle tone-burst pulse over an operating frequency range from 100 to 250 kHz, and an electromagnet power unit, as shown in Figure 4a. The electromagnet used to provide the pseudostatic magnetic field (Figure 4a) was in the form of the E-shaped ferrite core, as shown earlier in Figure 2a. It offered the capability of generating both an out-of-plane quasi-static field under the central pole region, and an in-plane field between the central and side poles. The measurements indicated that the generated quasi-static magnetic field ranged from 0 to 0.5 T under the central pole (out-of-plane), and from 0 to 1.1 T between the poles (in-plane), i.e., close to that expected from the COMSOL model. The racetrack coil shown earlier in Figure 3 was connecting to a high-power current pulse generator, delivering two levels of power at 450 W and 1.8 kW to generate the higher frequency dynamic field that generated the ultrasonic waves. Typical outputs from these two pulsing systems are shown in Figure 4b, and were those provided by the control system of the Innerspec unit, noting that the real current signals may have been different to this. Note that the frequency of the tone-burst input into the rf coil could be adjusted to examine the frequency response of the patch under various static field conditions.

**Figure 5:**
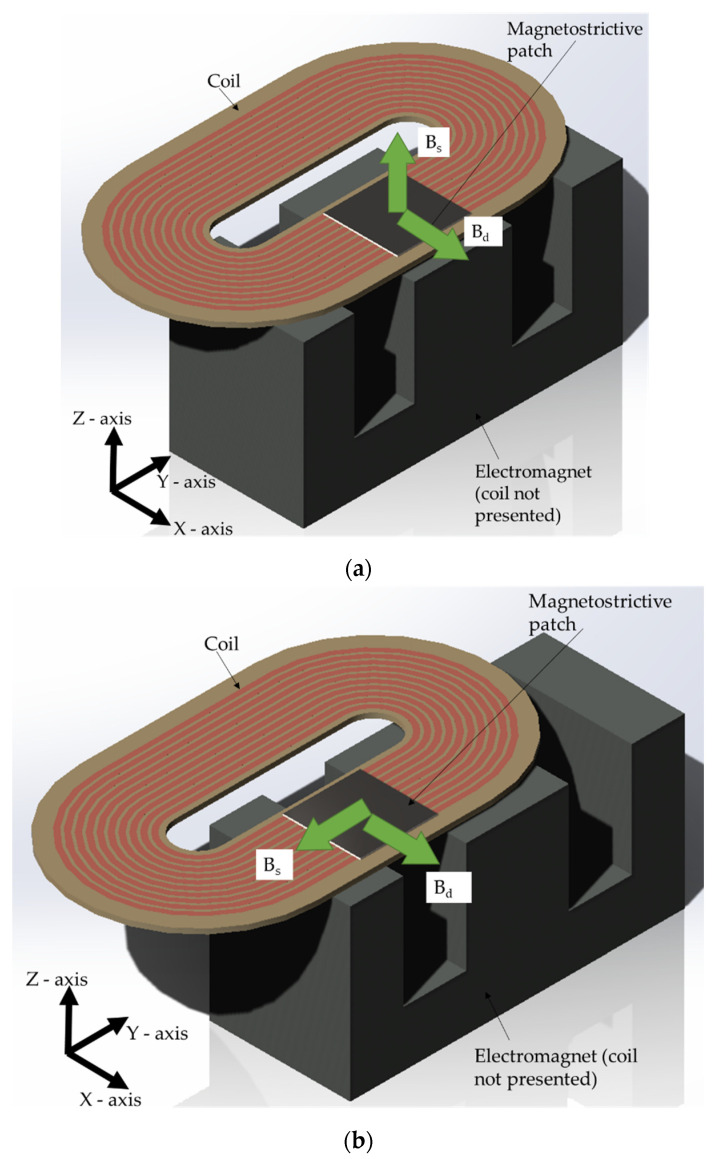
(**a**) Relative positions of the magnetostrictive patch, the coil and the electromagnet to generate the first set of experiments where the static magnetic field was out-of-plane while the dynamic magnetic field was in plane. (**b**) Relative positions of the magnetostrictive patch, the coil and the electromagnet to generate the second set of experiments where both the static magnetic field and the dynamic magnetic field were in-plane.

The dynamic displacement of the magnetostrictive patch was measured using a scanning vibrometer Polytec CLV 3000 3D (Polytec GmbH, Waldbronn, Germany), which could be used to detect both in-plane and out-of-plane motion. The laser system included three separate Doppler vibrometer heads, detecting ultrasonic motion in the three orthogonal axes (*X*, *Y* and *Z*, as shown in Figure 5) from the laser light reflected from the surface of the patch. 

Note that the output of the vibrometer in each case was a waveform of the ultrasonic particle velocity at each point of measurement. The 5-cycle tone-burst shown in Figure 4b was delayed via the triggering gate system to coincide with the time at which the field from the electromagnet reached a constant and maximum value, as indicated in Figure 4b. This ensured that the field from the electromagnet could be considered to be static, i.e., constant for each measurement. The patch was placed under the linear section of the racetrack coil, as shown earlier in Figure 3.

## 3. Results

### 3.1. Static Magnetic Field (B_s_) in the Out-of-Plane Direction

The experiment arrangement described above allowed the response of the magnetostrictive patch, as measured by the vibrometer as a function of frequency (f) and magnetic field direction. The first set of tests had the static field B_s_ in the out-of-plane (Z) direction while the dynamic field B_d_ was in-plane and predominantly in the *X* direction, as shown in Figure 5. The amplitude of the static field B_s_ and the dynamic coil excitation frequency f were then changed, and the ultrasonic vibration signal of the patch surface was recorded for the three different directions of motion (*X*, *Y* and *Z*) using the vibrometer. The amplitude of the dynamic field B_d_ was kept constant during these experiments. The frequency f of the current through the rf coil was varied from 100 to 240 kHz in 10 kHz steps, while the amplitude of B_s_ was varied from 0.1 to 0.5 T, with an increasing step of 0.05 T. 

**Figure 6:**
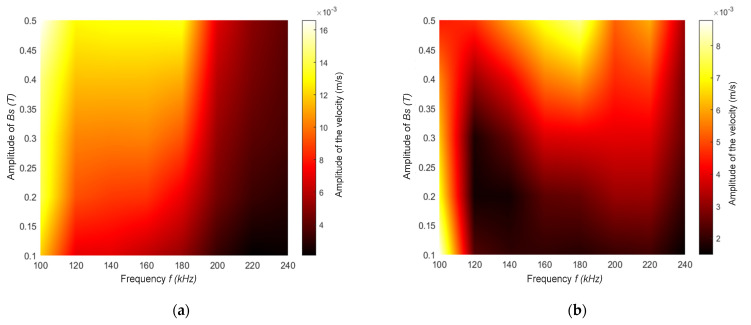

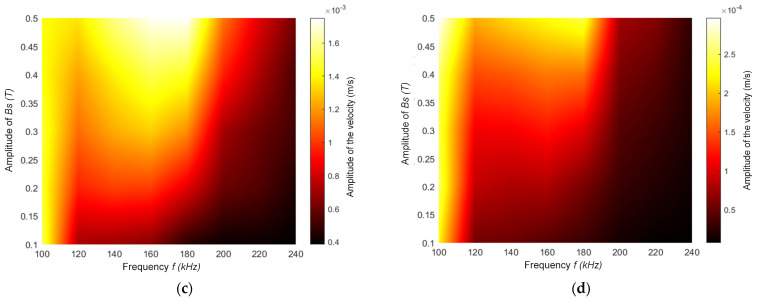
Particle velocity amplitude mapping while varying the static magnetic field amplitude and the frequency of the coil excitation in (**a**) the *X* direction, (**b**) the *Y* direction and (**c**) the *Z* direction. (**d**) The resultant magnitude was calculated using Equation (6).

The results of these experiments are presented in Figure 6a–c, which shows changes in the vibrational (particle velocity) amplitudes in the three orthogonal directions (*X*, *Y*, *Z*), respectively, as detected by the laser vibrometer. The resultant magnitude of the velocity, shown in Figure 6d, was calculated using the equation below:

(6) $$Magnitude\;Mag_{V}=\sqrt{V_{x}{}^{2}+V_{y}{}^{2}+V_{z}{}^{2}},$$

where *V_x_*, *V_y_* and *V_z_* are the particle velocity amplitudes in the directions *X*, *Y* and *Z*, respectively. Consider first the *X* direction (Figure 6a), where it can be seen that there was an abrupt reduction in vibration amplitude at a frequency of about 180 kHz. This change occurred at a lower frequency for smaller values of B_s_. Similar trends were seen in the *Z* direction (Figure 6c), but now a maximum in vibration amplitude was observed at B_s_ values above 0.4 T in the frequency range f = 140–180 kHz. In the *Y* direction (Figure 6b), a reasonably high amplitude existed for f = 140–200 kHz, but again only at higher B_s_ values. The overall magnitude of the vibration demonstrated that the most effective frequencies to use were those in the f = 140–200 kHz range, although relatively high vibrational levels could be obtained even for low frequencies with static magnetic field values below B_s_ = 0.2 T. Hence, an appropriate selection of the correct frequency and static field combination was required in order to generate a high amplitude ultrasonic signal, and this choice would vary depending on the direction of interest (e.g., in attempting to generate a particular ultrasonic wave mode in a sample to which the magnetostrictive material may be attached). 

This configuration led to the generation of Lorentz forces in the patch, mainly in the *X* direction. In addition, magnetostrictive forces were primarily expected to be generated in the *X* direction. From the figures below, it can be seen that both forces were constructively interfering. In fact, in Figure 6a, the amplitude of velocity was higher than the velocity amplitude recorded in the *Y* and *Z* directions. The amplitude of velocity in the *X* direction was double that compared to the velocity in the *Y* direction, and it was much higher than the amplitude of particle velocity vibrating in the *Z* direction. It can also be noticed that the vibrations were greater in the same direction as the dynamic magnetic field (B_d_). Preliminary modeling of the vibrating natural frequencies of the patch geometry identified that a vibrational mode of the patch geometry was oscillating at 100 kHz, which is in agreement with the results below. As a matter of fact, the variation of the frequency and the static magnetic field amplitude had minimal effect of the amplitude of the particle velocity. This was due to the fact that the patch was vibrating within the range of its natural frequency, which contributed to the overall vibration of the patch, compared to the Lorentz forces and the magnetostrictive forces. The mechanism had an effect on the generation of the guided waves, as their excitability was highly dependent on the driving frequency. In this case, the vibrations generating the guided waves were being induced mainly by the natural frequencies of the patch, rather than the effect of magnetostriction or Lorentz forces. 

### 3.2. B_s_ and B_d_ In-Plane, Parallel to the Surface of the Magnetostrcitve Patch and Orthogonal to Each Other 

**Figure 7:**
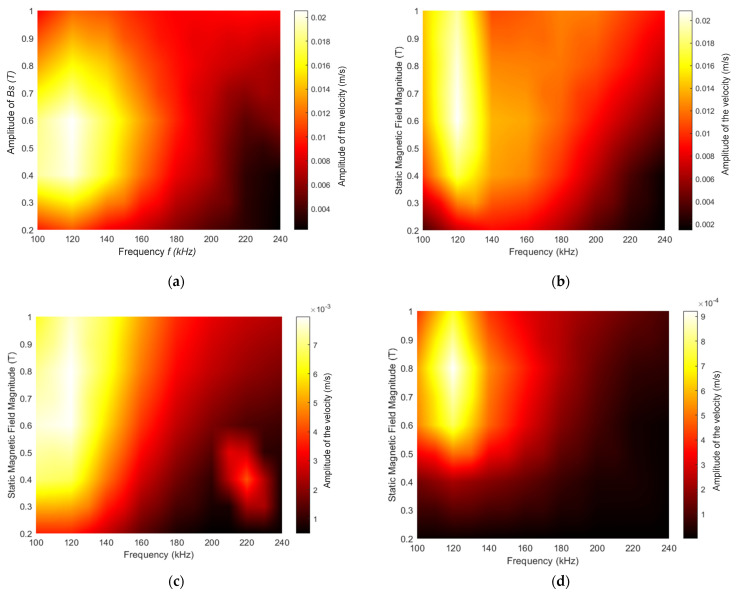
Velocity mapping while varying the static magnetic field B_s_ and the frequency in the (**a**) *X*, (**b**) *Y* and (**c**) *Z* directions, respectively. (**d**) The resultant magnitude of the particle velocity. The input power used to generate the dynamic field was 450 W throughout.

In this set of experiments, the B_d_ and B_s_ fields were in the *X* and *Y* direction, respectively (i.e., orthogonal to each other). Again, the resultant particle velocity amplitudes were recorded in the *X*, *Y* and *Z* directions as a function of f and B_s_. In addition, two amplitudes of the input power were used to generate the dynamic field B_d_ to investigate the effect of varying this parameter for a constant B_s_. In this configuration, the Lorentz force generation would be expected to be zero. The results for the case where the input power used to generate the dynamic field was 450 W are shown in Figure 7. Significant differences can be seen relative to Figure 6, where the field was out of plane. Now, the highest amplitudes in all three directions (*X*, *Y* and *Z*) occurred for lower frequencies of up to 40 kHz, and there was little signal at higher frequencies. The magnitude plot (Figure 7d) reflected these changes in the three different directions, in that the highest amplitude was achieved for specific combinations of frequency and applied static magnetic field amplitude. The magnitude was greatest at frequencies in the 105–140 kHz range, as expected from Figure 7a–c.

**Figure 8:**
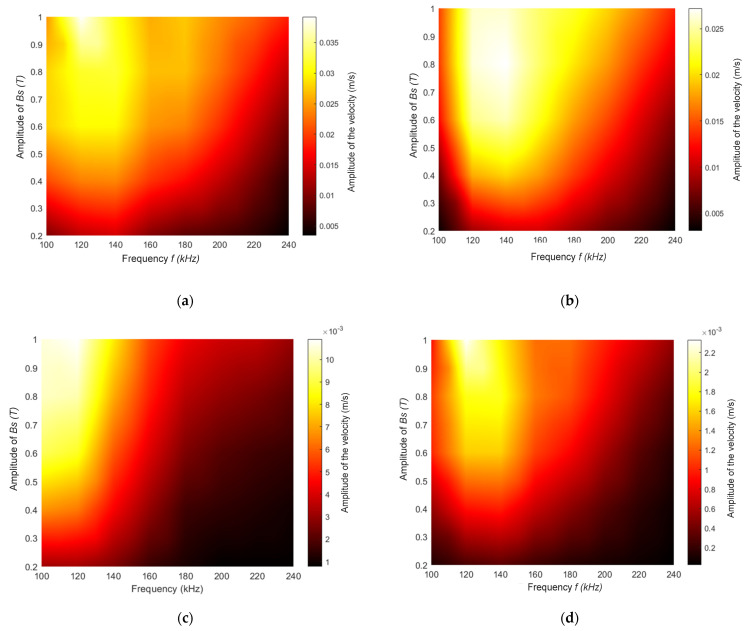
Velocity mapping while varying the static magnetic field B_s_ and the frequency in the (**a**) *X*, (**b**) *Y* and (**c**) *Z* directions, respectively. (**d**) A map of the resultant magnitude of the particle velocity. The input power used to generate the dynamic field was 1.8 kW throughout.

Results for the higher dynamic field power input of 1.8 kW are shown in Figure 8. It can be seen now that the distribution in amplitudes was spread over a wider range of frequencies than at the lower power level (Figure 7), with the maximum amplitudes being reached over the 120–160 kHz frequency range in the case of the *Y* direction. However, the maximum amplitudes in the *Z* direction were now located as before at frequencies below 120 kHz. As both magnetic fields were located parallel to the surface of the magnetostrictive patch, the vibration in the *Z* direction was mainly a result of the vibration in both *X* and *Y* directions, due to a Poisson ratio-type effect. Note also that the velocity amplitude recorded in the *X* direction had doubled from 0.02 to 0.04 m/s, while the velocity captured in the *Y* direction increased by 50% (from 0.02 to 0.03 m/s). 

From Figure 7, it can still be seen that the vibration velocity amplitude remained relatively high at around 100 kHz, indicating that by adopting this design, the vibration due to the natural frequency was still significant and could influence the effects of the Lorentz forces and the magnetostrictive forces. It is worth mentioning that the vibrations due to the natural frequencies of the patch could be selected, as in an NDT application the patch will be glued, which leads to a variation of the overall natural frequency of the system. 

Another important observation can be obtained by comparing the results from the situation where the external static field was applied either out-of-plane (Figure 6) or parallel to the surface of the magnetostrictive patch (Figure 7 and Figure 8). In fact, the magnitude of the vibration had increased by almost an order of magnitude (from 2.5 × 10^−4^ to 2.2 × 10^−3^ m/s), and the working region had increased to provide more combinations of the frequency/static magnetic field amplitudes, at which a reasonable response was observed. Although the boundary conditions of the patch had changed between both experiments, when the magnetostrictive patches were used to generate guided waves for inspection purposes, the variation of the magnetic field directions and amplitude could enhance the signal generation and increase detectability of defects. 

Compared to the previous experiments, by using high power with both magnetic fields placed in-plane, the vibration of the patch might be expected to be larger. In fact, the patch vibration pattern was similar to the magnetostriction curve of the ferromagnetic material. The vibration amplitude increased when a high static magnetic field amplitude was applied. The vibration velocity was inversely proportional to the frequency, and the patch was vibrating at high amplitudes within the low-frequency range. 

**Figure 9:**
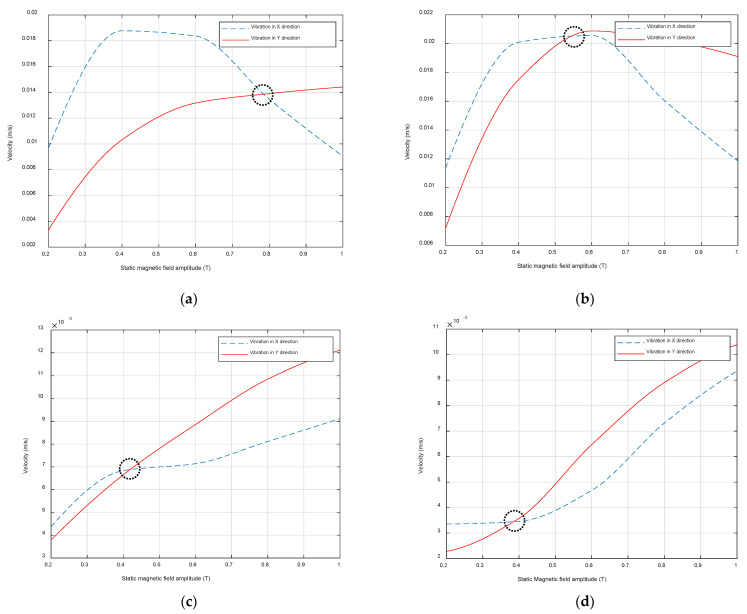
Magnetostrictive patch vibration in the *X* and *Y* directions as a function of static magnetic field amplitude for (**a**) 100, (**b**) 120, (**c**) 200 and (**d**) 220 kHz.

Another set of experiments examined the relative amplitudes of the static (B_s_) and dynamic (B_d_) fields for particular fixed values of dynamic rf excitation frequency (f). Here, the amplitudes of vibration in the *X* and *Y* directions were captured while varying B_s_ for a fixed dynamic field amplitude B_d_ of 1.8 kW. The two fields were orthogonal to each other to minimize Lorentz force generation, as the study on magnetostriction generation was of interest. The results are shown in Figure 9a–d for frequencies of 100, 120, 200 and 220 kHz, respectively.

As can be seen in Figure 9, the amplitudes in both directions showed nonlinear behavior with increasing static magnetic field amplitude, and this confirmed the results from the previous set of experiments. For relatively low frequencies, it was observed that the amplitude of the velocity in the *Y* direction saturated when applying a static magnetic field in the range from 0.4 to 0.6 T, with the amplitude of the velocity decreasing at higher values. Conversely, when high frequencies were selected to drive the dynamic magnetic field applied to the patch, the velocity in both *X* and *Y* directions had similar trends, as the amplitude increased when the applied static magnetic field was increased. This trend was similar to the magnetostrictive curve measured by applying a static magnetic field to establish the properties of the ferromagnetic material. It is also important to mention that while increasing the static magnetic field, the amplitude of the velocity in the *Y* direction surpassed the amplitude of the velocity in the *X* direction. The “switching” point value (circled in Figure 9 in each case) decreased as the frequency increased. In fact, the amplitude in the *Y* direction became higher than in the *X* direction more quickly when increasing the oscillation frequency.

## 4. Discussion

It was evident that the relative values of B_d_ and B_s_ had a significant effect on the generation of magnetostriction within a thin magnetostrictive patch. First, the magnetic field generated through the electromagnet was applied in the out-of-plane direction compared to the magnetostrictive patch surface, while the dynamic magnetic field direction was in-plane. The results showed that by modifying the frequency and the amplitude of the static magnetic field, the vibration generated within the patch could be controlled and enhanced. The analysis showed a complex link between rf coil frequency and the static magnetic field amplitude. For the case where the frequency was around 100 kHz, the variation of the applied magnetic field had a small effect on the vibration amplitude in all directions. At higher frequencies, careful selection of the frequency and the static field amplitude was required for this configuration, as the enhancement of the signal was not achieved by simply increasing the static magnetic field amplitude. This could be seen for frequencies such as 130 kHz and at frequencies above 200 kHz. 

The second set of experiments consisted of placing both B_d_ and B_s_ in-plane, with the two fields orthogonal to each other, so as to minimize the generation of Lorentz forces in the patch, allowing magnetostriction effects to be studied in isolation. The power energizing the driving coil was also set to two different values (450 W and 1.8 kW). In all cases, a more complex interaction was observed. With a driving power of 450 W into the dynamic coil, the performance of the patch was optimum only over certain frequency and static magnetic field ranges. This could be due to the fact that the energy levels used to generate the magnetostrictively induced vibrations were relatively low. The static magnetic field (B_s_) acted to align the magnetic domains in the direction of B_s_. The modulation provided by B_d_ was superimposed on this, and when the power induced was relatively low, the effect on the rotation of the magnetic domains was very limited. It should be noted that the magnitude of vibration in all directions was enhanced and, in some cases doubled, compared to the out-of-plane configuration. This was due to the fact that thin magnetostrictive films were used. Increasing the power energizing the dynamic coil to 1.8 kW showed a more consistent trend. Increasing the amplitude of the dynamic field enhanced velocity amplitudes in the *X* direction and the *Y* direction. It was also observed that higher velocities could be achieved at higher drive frequencies, in this case at 240 kHz. In fact, by increasing the input power, the energy applied to the magnetostrictive patch was increasing, which could facilitate a wider operational range. For higher levels of B_d_, the patch responded by generating higher acoustic particle velocity amplitudes at higher frequencies.

In addition, by placing both magnetic fields so that the generation of the Lorentz forces was minimized, a switching point was observed when varying the amplitudes of the magnetic fields (Figure 9). For the low-frequency range (from 100 to 220 kHz), a higher level of B_s_ was required so that the dominant vibrations were aligned with the *Y* direction. The switching point between the magnitudes of vibration in the *X* and *Y* directions moved to a lower frequency when the frequency was increasing. In fact, for higher frequencies, the energy induced by the dynamic magnetic field was decreasing, which was leading to the situation where the vibration of the magnetic domains was dominated by the static magnetic field applied in the *Y* direction.

## 5. Conclusions

By using the laser vibrometer system, the vibration of a thin magnetostrictive patch when subject to variable experimental parameters was analyzed and studied. Interesting observations were identified by varying the static magnetic field direction and amplitude, the dynamic magnetic field or the excitation frequency. It was demonstrated that by using the same patch and modifying the excitation parameters, the signal generated from the patch through the magnetostriction phenomena could be enhanced, and the patch was able to generate higher vibration velocities and ultrasonic wave amplitudes. The amplitude of the vibration could be altered by simply modifying the direction or amplitude of B_d_ and B_s_. In previous work, the frequency effect on magnetostriction was not well-established, as the effect was only studied by applying a static magnetic field, without considering the effect of the application of an oscillating magnetic field. It was shown that by placing the static magnetic field in the out-of-plane direction and the selection of low operating frequencies, the variation of the static magnetic field was minimal, but that the effect was significant when the same field was in-plane. By further increasing the amplitude of the dynamic field as well as the static magnetic field, the performance of the thin magnetostrictive patch could be enhanced and controlled. A more global understanding and investigation of the effect of these parameters will be of interest to the NDE research community, as such patches can be widely used to overcome multiple limitations imposed by the use of piezoelectric transducers for applications such as guided-wave inspection.

## Figures and Tables

**Table 1:** Patch design parameters.

Patch Material	Iron–Cobalt Alloy
Shape	square
Dimensions	20 mm × 20 mm × 0.1 mm
Young’s Modulus	200 GPa
Poisson Ratio	0.29
Density	8.12 g/cm^3^
Electrical Resistivity	0.42 µΩm
Permeability	18,000 N A^−2^
Saturation Magnetostriction	70 ppm
Saturation Magnetisation	2.35 T

## Data Availability

Not Applicable.

## References

[1] Zhu W., Bian L.X., Cheng L., Rui X.T. (2017). Non-linear compensation and displacement control of the bias-rate-dependent hysteresis of a magnetostrictive actuator. Precis. Eng..

[2] Bieńkowski A., Kulikowski J. (1980). The magneto-elastic Villari effect in ferrites. J. Magn. Magn. Mater..

[3] Witthauer A., Kim G., Faidley L., Zou Q., Wang Z. (2014). Design and characterization of a flextensional stage based on Terfenol-D actuator. Int. J. Precis. Eng. Manuf..

[4] Li M., Li J., Bao X., Mu X., Gao X. (2017). Magnetostrictive Fe_82_Ga_13.5_A_l4_._5_ wires with large Wiedemann twist over wide temperature range. Mater. Des..

[5] Chang H., Liao S., Hsieh H., Wen J., Lai C., Fang W. (2016). Magnetostrictive type inductive sensing pressure sensor. Sens. Actuators A Phys..

[6] Bieńkowski A., Szewczyk R. (2018). Magnetostrictive Properties of Mn_0.70_Zn_0.24_Fe_2.06_O_4_ Ferrite. Materials.

[7] Zhukov A., Ipatov M., Churyukanova M., Talaat A., Blanco J.M., Zhukova V. (2017). Trends in optimization of giant magnetoimpedance effect in amorphous and nanocrystalline materials. J. Alloys Compd..

[8] Ghosh D. (2006). Structural Health Monitoring of Composite Structures Using Magnetostrictive Sensors and Actuators. Ph.D. Thesis.

[9] Joule J.P. (1847). On the effects of magnetism upon the dimensions of iron and steel bars. Phil. Mag..

[10] Hirao M., Ogi H. (2003). EMATs for Science and Industry: Noncontacting Ultrasonic Measurements.

[11] Jiles D. (2015). Introduction to Magnetism and Magnetic Materials.

[12] Seco F., Martín J.M., Jiménez A.R. (2008). Improving the accuracy of magnetostrictive linear position sensors. IEEE Trans. Instrum. Meas..

[13] Li Y., Sun L., Jin S., Sun L.B. Development of magnetostriction sensor for on-line liquid level and density measurement. Proceedings of the 2006 6th World Congress on Intelligent Control and Automation.

[14] Gao X., Pei Y., Fang D. (2008). Magnetomechanical behaviors of giant magnetostrictive materials. Acta Mech. Solida Sin..

[15] Sablik M.J., Jiles D.C. (1988). A model for hysteresis in magnetostriction. J. Appl. Phys..

[16] Butler J.L. (1988). Application Manual for the Design of Terfenol-D Magnetostrictive Transducers.

[17] Liang X., Dong C., Chen H., Wang J., Wei Y., Zaeimbashi M., He Y., Matyushov A., Sun C., Sun N. (2020). A review of thin-film magnetoelastic materials for magnetoelectric applications. Sensors.

[18] Hall D.L., Flatau A.B. (1995). One-dimensional analytical constant parameter linear electromagnetic-magnetomechanical models of a cylindrical magnetostrictive (Terfenol-D) transducer. J. Intell. Mater. Syst. Struct..

[19] Li P., Liu Q., Li S., Wang Q., Zhang D., Li Y. (2017). Design and numerical simulation of novel giant magnetostrictive ultrasonic transducer. Results Phys..

[20] Zhai G., Jiang T., Kang L., Wang S. (2010). Minimizing influence of multi-modes and dispersion of electromagnetic ultrasonic Lamb waves. IEEE Trans. Ultrason. Ferroelectr. Freq. Control.

[21] Moffett M.B., Clark A.E., Wun-Fogle M., Linberg J., Teter J.P., McLaughlin E.A. (1991). Characterization of Terfenol-D for magnetostrictive transducers. J. Acoust. Soc. Am..

[22] Gińko O., Juś A., Szewczyk R. (2016). Test Stand for Measuring Magnetostriction Phenomena under External Mechanical Stress with Foil Strain Gauges. International Conference on Automation.

[23] Weiss P. (1907). L’hypothèse du champ moléculaire et la propriété ferromagnétique. J. Phys. Theor. Appl..

[24] Kim Y.Y., Kwon Y.E. (2015). Review of magnetostrictive patch transducers and applications in ultrasonic nondestructive testing of waveguides. Ultrasonics.

[25] Dapino M.J. (2004). On magnetostrictive materials and their use in adaptive structures. Struct. Eng. Mech..

[26] Joule J.P. (1842). On a new class of magnetic forces. Ann. Electr. Magn. Chem..

[27] Dimitropoulos P.D., Avaritsiotis J.N. (2001). A micro-fluxgate sensor based on the Matteucci effect of amorphous magnetic fibers. Sens. Actuators A Phys..

[28] Villari E. (1865). Ueber die Aenderungen des magnetischen Moments, welche der Zug und das Hindurchleiten eines galvanischen Stroms in einem Stabe von Stahl oder Eisen hervorbringen. Ann. Phys..

[29] Zitoun A., Dixon S., Edwards G., Hutchins D. (2020). Experimental Study of the Guided Wave Directivity Patterns of Thin Removable Magnetostrictive Patches. Sensors.

[30] Laguerre L., Aime J., Brissaud M. (2002). Magnetostrictive pulse-echo device for non-destructive evaluation of cylindrical steel materials using longitudinal guided waves. Ultrasonics.

[31] Kim Y., Moon H., Park K., Lee J. (2011). Generating and detecting torsional guided waves using magnetostrictive sensors of crossed coils. NDT E Int..

[32] Juś A., Nowak P., Gińko O. (2017). Assessment of the magnetostrictive properties of the selected construction steel. Acta Phys. Pol. A.

[33] Wu J., Tang Z., Yang K., Lv F. (2019). Signal strength enhancement of magnetostrictive patch transducers for guided wave inspection by magnetic circuit optimization. Appl. Sci..

[34] Lee E.W. (1958). Magnetostriction curves of polycrystalline ferromagnetics. Proc. Phys. Soc..

[35] Hu C., Xu J. (2019). Center frequency shift in pipe inspection using magnetostrictive guided waves. Sens. Actuators A Phys..

[36] Kim H.J., Lee J.S., Kim H.W., Lee H.S., Kim Y.Y. (2014). Numerical simulation of guided waves using equivalent source model of magnetostrictive patch transducers. Smart Mater. Struct..

